# Exploring Brain Structural and Functional Biomarkers in Schizophrenia *via* Brain-Network-Constrained Multi-View SCCA

**DOI:** 10.3389/fnins.2022.879703

**Published:** 2022-06-20

**Authors:** Peilun Song, Yaping Wang, Xiuxia Yuan, Shuying Wang, Xueqin Song

**Affiliations:** ^1^School of Information Engineering, Zhengzhou University, Zhengzhou, China; ^2^Department of Psychiatry, The First Affiliated Hospital of Zhengzhou University, Zhengzhou, China; ^3^Biological Psychiatry International Joint Laboratory of Henan/Zhengzhou University, Zhengzhou, China

**Keywords:** multimodal brain image analysis, brain network constraint, sparse canonical correlation analysis, schizophrenia, biomarker

## Abstract

Recent studies have proved that dynamic regional measures extracted from the resting-state functional magnetic resonance imaging, such as the dynamic fractional amplitude of low-frequency fluctuation (d-fALFF), could provide a great insight into brain dynamic characteristics of the schizophrenia. However, the unimodal feature is limited for delineating the complex patterns of brain deficits. Thus, functional and structural imaging data are usually analyzed together for uncovering the neural mechanism of schizophrenia. Investigation of neural function-structure coupling enables to find the potential biomarkers and further helps to understand the biological basis of schizophrenia. Here, a brain-network-constrained multi-view sparse canonical correlation analysis (BN-MSCCA) was proposed to explore the intrinsic associations between brain structure and dynamic brain function. Specifically, the d-fALFF was first acquired based on the sliding window method, whereas the gray matter map was computed based on voxel-based morphometry analysis. Then, the region-of-interest (ROI)-based features were extracted and further selected by performing the multi-view sparse canonical correlation analysis jointly with the diagnosis information. Moreover, the brain-network-based structural constraint was introduced to prompt the detected biomarkers more interpretable. The experiments were conducted on 191 patients with schizophrenia and 191 matched healthy controls. Results showed that the BN-MSCCA could identify the critical ROIs with more sparse canonical weight patterns, which are corresponding to the specific brain networks. These are biologically meaningful findings and could be treated as the potential biomarkers. The proposed method also obtained a higher canonical correlation coefficient for the testing data, which is more consistent with the results on training data, demonstrating its promising capability for the association identification. To demonstrate the effectiveness of the potential clinical applications, the detected biomarkers were further analyzed on a schizophrenia-control classification task and a correlation analysis task. The experimental results showed that our method had a superior performance with a 5–8% increment in accuracy and 6–10% improvement in area under the curve. Furthermore, two of the top-ranked biomarkers were significantly negatively correlated with the positive symptom score of Positive and Negative Syndrome Scale (PANSS). Overall, the proposed method could find the association between brain structure and dynamic brain function, and also help to identify the biological meaningful biomarkers of schizophrenia. The findings enable our further understanding of this disease.

## Introduction

Schizophrenia (SCZ) is a severe psychiatric disorder, which is characterized by cognitive dysfunction, delusions, hallucinations, and personality disturbance (Ventura et al., 2009). It has affected about 1% of the population throughout the world, and has potentially become a lifetime burden for the patients and their families (McGrath et al., 2008). Finding objective biomarkers for the accurate diagnosis and effective intervention in the early stage of SCZ is of great importance for the neuroscience and medical science. However, it is still challenging to identify the accurate biomarkers of SCZ as the pathological mechanism of this disease is unclear yet (Insel, 2010). In the recent decades, the advancements in magnetic resonance imaging (MRI) techniques have provided an alternative opportunity to search for SCZ-related biomarkers. Using the non-invasive MRI, such as functional MRI (fMRI), structural MRI (sMRI), and diffusion tensor imaging (DTI), the brain functional and structural abnormalities can be detected, facilitating the understanding about the pathophysiology of SCZ (Ding et al., 2019; Steardo et al., 2020,Sagarwala and Nasrallah, 2021).

A lot of literatures proved that fMRI has played an important role in the analysis of SCZ (Wang et al., 2018; Steardo et al., 2020). Based on the resting-state fMRI (rs-fMRI), the static functional measures are commonly extracted to find the abnormal patterns in brain, and then, the disease-related biomarkers are identified for further analysis. Currently, beyond the traditional static analysis of functional brain activity, the temporal dynamic features of brain have attracted more and more attention, which can depict the temporal alteration of brain function (Filippi et al., 2019). Dynamic regional measurements at resting-state were widely investigated on brain disorders, demonstrating their sensitive detection capability for the abnormal characteristics of brain (Tang et al., 2018). Dynamic fractional amplitude of low-frequency fluctuation (d-fALFF) is one of the popularly used dynamic regional measurements in SCZ research, which can reflect the temporal variability of the amplitude of intrinsic neural activity (Yan et al., 2017; Zhang et al., 2019). However, as a complex brain disorder, such single-modality data cannot adequately depict the defective pattern caused by SCZ. Recently, an increasing number of evidences have shown that the combination of multimodal imaging data might provide distinct and complementary information, contributing to the comprehensive investigation of SCZ (Zhuang et al., 2019; Lei et al., 2020a). Among these multimodal studies, the fMRI and sMRI were most commonly combined, following with a machine learning method, to conduct the subsequent analysis such as the classification of healthy controls (HCs) and SCZ (Cao et al., 2020). Even though the improved performances were obtained based on these multimodal methods, the inter-modality relationships were inevitably overlooked in most of these studies, which are also important for the multimodal analysis.

In the neuroscience field, researchers have been aware of the importance of exploring the inter-modality associations (Du et al., 2020). Various types of correlation analysis method have been proposed to identify the relationship between different modalities (Shen and Thompson, 2020). Within them, the sparse canonical correlation analysis (SCCA) is one of the most popular methods (Witten et al., 2009). The SCCA could identify multivariate associations between two sets of variables, while it is an unsupervised approach, indicating that it cannot utilize the diagnosis information to guide the exploration of disease-related associations. So, it is limited to find the disease-related and biologically interpretable biomarkers. To overcome this shortcoming, the multi-view SCCA (e.g., three-view SCCA) was adopted by including the diagnosis information as the third type of data, with the aim of simultaneously maximizing the pairwise correlations among diagnosis information and other two sets of variables. By introducing the diagnosis information, the disease-related biomarkers could be detected based on this kind of methods (Hao et al., 2017; Won et al., 2020). However, from the point of view of biologically meaningful interpretation, it still remains a challenge to obtain biologically interpretable findings for the current multi-view SCCA method. To incorporate the biologically meaningful structure knowledge, simplify the model complexity, and reduce the risk of overfitting, different regularization methods were used in the SCCA, such as lasso penalty (Witten et al., 2009), graph-constrained elastic net (Kim et al., 2021), group lasso regularization (Du et al., 2014), and so on. Recent studies have tried to associate the detected brain regions with a certain brain network, and found network-level aberrant alterations in SCZ (Li et al., 2019; Supekar et al., 2019). Based on the observations above, it is hypothesized that this brain-network-based structural information might be helpful for the exploration of SCZ-related biomarkers. To our best knowledge, this kind of structure information has not been utilized in SCCA yet. Thus, a novel multi-view SCCA method, which could simultaneously utilize the brain-network-based structural information and the diagnosis information to help to explore the disease-related biomarkers, is needed for better identifying disease-related multivariate associations and producing biologically meaningful findings.

In this study, we proposed a novel brain-network-constrained multi-view SCCA (BN-MSCCA) to explore the complex relationships between brain structure and dynamic brain function, and subsequently identify the SCZ-related biomarkers. The temporal dynamic analysis (TDA) was first performed to compute the dynamic brain functional measurement (e.g., d-fALFF) using the sliding window method. Then, voxel-based morphometry (VBM) analysis was performed to obtain the gray matter (GM) map. After that, the region of interest (ROI)-based features were further extracted from these two measurements. Finally, the three-view canonical correlation analysis jointly with the diagnosis information was performed. Moreover, the brain-network-based structural constraint was introduced into the model to prompt the detected biomarkers more interpretable. Using 191 SCZ and 191 HC data, BN-MSCCA obtained more sparse canonical weight patterns and higher canonical correlation coefficients (CCCs). The subsequent classification and PANSS correlation experiments also proved the capability of detected biomarkers for depicting the abnormalities of SCZ.

The rest of this article is organized as follows. Section Materials and Methods describes the materials used in this study and the proposed BN-MSCCA method following with its optimization algorithm. The specific experimental settings and the results are introduced in Section Experiments and Results. In Section Discussion, a comprehensive discussion about the results is presented. Section Conclusion concludes this article.

## Materials and Methods

**Figure 1:**
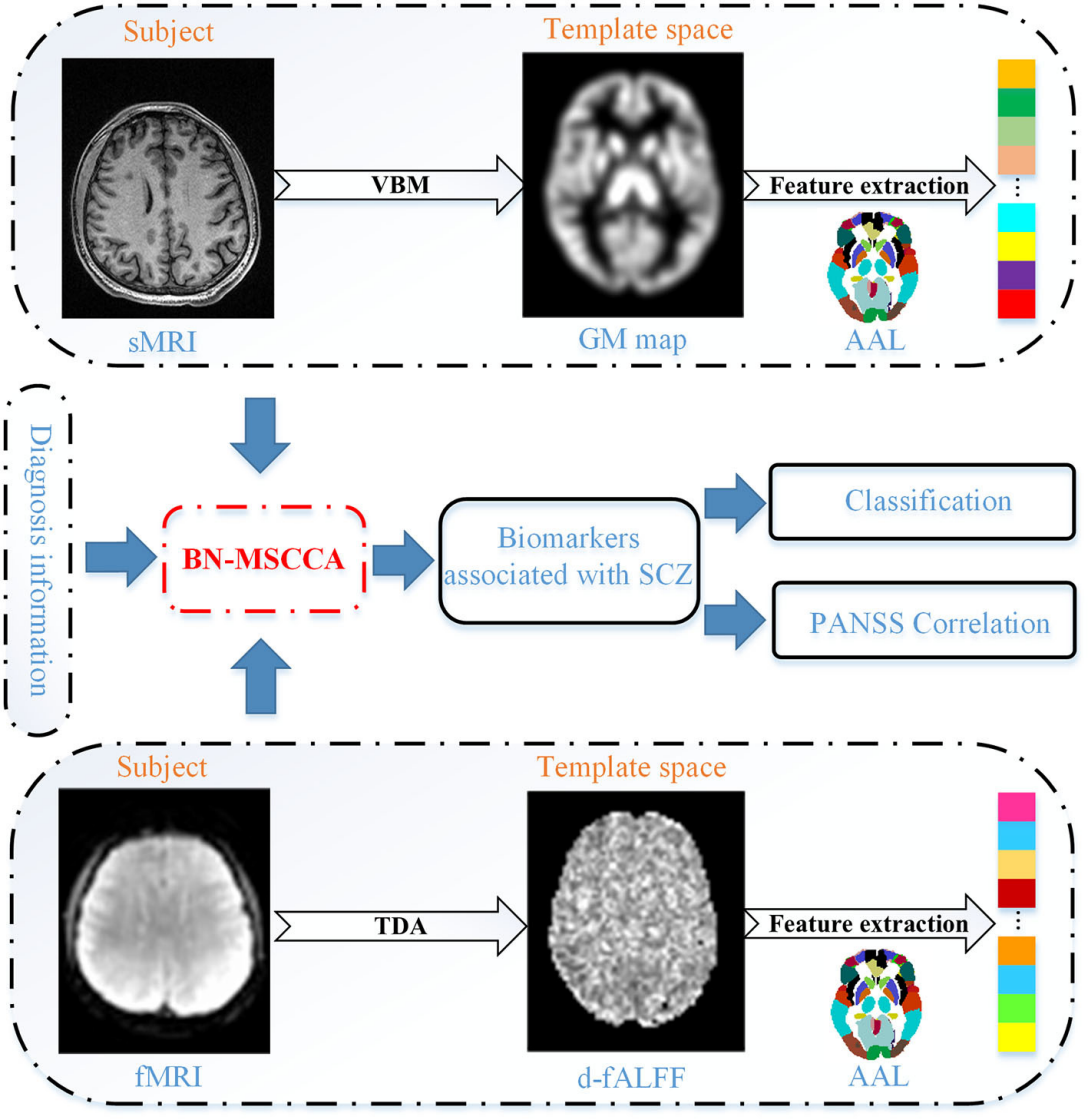
The flowchart of our proposed method.

The proposed method comprised of four main steps, such as (1) data preprocessing and feature extraction, (2) identifying associations using the proposed BN-MSCCA method, (3) detecting SCZ-related biomarkers, and (4) subsequent analysis based on the detected biomarkers. Figure 1 presents the overall flowchart of the proposed BN-MSCCA method. In this section, we mainly introduce the data preprocessing, feature extraction, the proposed BN-MSCCA method, and its optimization algorithm.

### Data Preprocessing and Feature Extraction

#### Data Acquisition

The dataset used in this study was collected at the First Affiliated Hospital of Zhengzhou University, Zhengzhou, China. For the patients with SCZ, the psychiatric diagnoses were based on the USA manual of the Diagnostic and Statistical Manual of Mental Disorders IV (DSM-IV) (Guze, 1995). The exclusion criteria included the presence of traumatic brain injury, severe endocrine diseases, anemia, hematological diseases or other mental diseases, a history of excessive drinking or abuse of psychotropic substances, or the incompletion of MRI examination. Finally, 191 patients with SCZ were enrolled in this work, in which 108 patients have the complete PANSS score.

A total of 191 healthy subjects with matched age and gender were recruited as the HC group. The subjects were able to complete the MR scanning and had no history of organic brain disease or other chronic diseases and mental disorders, nor a family history of psychosis. The demographic information of the studied subjects is summarized in Table 1. The study procedures have passed the approval of the ethics committee of the First Affiliated Hospital of Zhengzhou University. All the participants and their legal guardian have consented and signed the informed consent.

**Table 1:** Participant demographics.

**Characteristic**	**SCZ (*N* = 191)**	**HC (*N* = 191)**	*p*-value*
Age (mean ± sd, year)	23.16 ± 8.45	23.28 ± 4.69	0.863
Gender (M/F)	91/100	89/102	0.838

* *t-test is used for comparison of age, and χ^2^ test is used for gender comparison*.

The T1-weighted MRI and rs-fMRI were acquired on a GE Discovery 750 3T MRI scanner with an 8-channel head coil. The T1 images were acquired with repetition time (TR) = 8.2 ms, echo time (TE) = 3.2 ms, field of view (FOV) = 256 mm × 256 mm, slice number = 188, slice thickness = 1 mm, flip angle = 12°, and 256 × 256 matrix. The rs-fMRIs were collected using the echo planar imaging sequence (EPI) with TR = 2,000 ms, TE = 30 ms, FOV = 220 mm × 220 mm, slice number = 32, slice thickness = 4 mm, inter-slice gap = 0.5 mm, flip angle = 90°, and 64 × 64 matrix, and the scanning time for each subject is about 6 min (resulting in 188 volumes). During the scanning, the participants were required to think about nothing in particular and keep their head still and eyes closed at the same time.

#### Data Pre-processing

CAT12 (http://www.neuro.uni-jena.de/cat), an extension toolkit of SPM12, provides a platform for both surface-based morphometry and VBM analysis. Using the T1-weighted MRI data, we followed the standard pipeline of the CAT12 to conduct the VBM analysis. The main steps included the correction of bias-field inhomogeneities, segmentation of brain tissues (gray matter, white matter and cerebrospinal fluid), spatial normalization into the Montreal Neurological Institute (MNI) space, resampling to 1.5 mm × 1.5 mm × 1.5 mm, and non-linear modulation. Finally, the obtained GM maps were smoothed using an 8 mm full width at half maximum (FWHM) Gaussian kernel.

The rs-fMRI data were preprocessed using the DPABI (http://www.rfmri.org/dpabi) software. The processing steps are as follows. First, the initial 10 volumes were removed, followed by the slice-timing correction. Then, the time series of each subject were realigned by a linear transformation. After the realignment, the mean functional image was co-registered to the corresponding T1 image, which had been segmented into gray matter, white matter, and cerebrospinal fluid using a unified segment method (http://www.fil.ion.ucl.ac.uk/spm). Finally, the functional images were resampled to 3 mm × 3 mm × 3 mm and then normalized into the MNI space using the DARTEL (Ashburner, 2007). To alleviate the influence of noise, the images were smoothed by a 4-mm FWHM Gaussian kernel and band-pass filtered within 0.01–0.1 HZ. The nuisance regression was used to regress out the irrelevant variable interferences, including the Friston-24 parameters, white matter signal, cerebrospinal fluid signal, and global signal.

#### Feature Extraction

Based on the GM images, the ROI-based features were extracted for the subsequent analysis. First, the normalized and modulated GM maps were resampled to 3 mm × 3 mm × 3 mm using the trilinear method. Then, the whole GM map was divided into 116 ROIs according to the AAL atlas. Finally, we averaged the values within each ROI to obtain the ROI-based measurements.

As for the rs-fMRI, we computed the d-fALFF through the TDA module in DPABI. First, we divided all BOLD time series of the whole brain into multiple overlapping windows. In this study, we empirically set the width of each sliding window as 60 s and the interval between time windows as 10 s. Second, the fALFF was calculated based on the time series in a specific time window. The time series was converted to the frequency domain using the fast Fourier transform, and the square root of power spectrum was computed. Then, the sum of amplitude in 0.01–0.1 HZ was divided by the entire frequency range to obtain the fALFF map. Subsequently, the mean and standard deviation of each voxel in the fALFF maps of all sliding windows were computed. Finally, we obtained the coefficient of variation (CV) of these fALFF maps, which was usually regarded as d-fALFF and it was acquired by dividing the standard deviation by the mean in details. The raw d-fALFF of each voxel was further divided by the mean value of the whole brain to reduce the global effects of variability across the subjects (Wang et al., 2020). Similar to T1 image, the d-fALFF of each ROI was also obtained based on the AAL template. To remove the possible effects of age and gender, we pre-adjusted all these imaging features using the regression.

### Methods

In this article, we define a matrix using the uppercase letter and a vector using the lowercase letter. Specifically, let *X* ∈ ℝ^*n*×*p*^ and *Y* ∈ ℝ^*n*×*q*^ represent the data matrices, where *X* corresponds to the d-fALFF-based features with *n* samples and *p* variables, and *Y* corresponds to the GM volume-based features with *n* samples and *q* variables.

#### SCCA

For identifying the complex multivariate associations, SCCA was proposed with the aim to find the linear transformation of *X* and *Y* and obtain the maximal correlation between these two transformed variables. Meanwhile, the penalty terms were introduced to make the variables more sparse and avoid the overfitting (Witten et al., 2009). The SCCA could be formulated as follows.


(1) $$\underset{u,v}{max}u^{T}X^{T}Yv$$

$$\begin{array}{l} s.t.\;u^{T}X^{T}Xu\leq 1,\;{\;v}^{T}Y^{T}Yv\leq 1,\;{\;||u||}_{1}\leq a_{1},\;{\;||v||}_{1}\leq a_{2} \end{array}$$

*where u* and *v* are the canonical weights for the corresponding data modalities (*X* and *Y*), showing the contribution of each feature in this canonical correlation. In this model, the *u*^*T*^*X*^*T*^*Xu* ≤ 1 and *v*^*T*^*Y*^*T*^*Yv* ≤ 1 are used to describe the covariance structure of the data. The ||*u*||_1_ ≤ *a*_1_ and ||*v*||_1_ ≤ *a*_2_ are constraints for controlling the sparsity and selecting the most relevant features from the d-fALFF-based and GM volume-based features, respectively. However, the SCCA can only capture associations between two distinct types of data, which cannot meet the demand for identifying multi-view associations among more than two different types of modalities. On the other hand, the SCCA is an unsupervised method indicating that it cannot make full use of the diagnosis information.

#### Multi-View SCCA

Recently, to uncover the complex associations among multiple types of data, a variant of SCCA, called multi-view SCCA (MSCCA), was proposed to include more than two types of data (Witten and Tibshirani, 2009; Hao et al., 2017). Using the MSCCA, some studies were performed to investigate relationships among three modalities (Du et al., 2021). The MSCCA could be formulated as follows:


(2) $$\underset{u,v,w}{max}u^{T}X^{T}Yv+v^{T}Y^{T}Zw+w^{T}Z^{T}Xu$$

$$\begin{array}{l} s.t.\;u^{T}X^{T}Xu\leq 1,\;{\;v}^{T}Y^{T}Yv\leq 1,{\;w}^{T}Z^{T}Zw\leq 1,\\ ||u|{|}_{1}\leq a_{1},\;{\;||v||}_{1}\leq a_{2},||w|{|}_{1}\leq a_{3} \end{array}$$

Note that *Z* ∈ ℝ^*n*×*r*^ is the third type of data, where *r* is its feature dimension and *w* is the canonical weight of *Z*. As a special case of MSCCA, the task-oriented MSCCA was used to incorporate the supervision information as the third type of data, which is from the target task (Hao et al., 2017; Won et al., 2020). According to these studies, the MSCCA has demonstrated its promising capacity for uncovering the disease-related biomarkers. However, the data structure information was overlooked in these methods as the L1-norm penalty can only enforce the individual sparsity without considering the internal structure of the data.

#### BN-MSCCA

In this work, we focused on association identification among the GM volume-based and d-fALFF-based features. The diagnosis information was also introduced into the model, so that we can find the brain functional and structural biomarkers that are relevant to the disease. Considering the brain structure information as prior information, we further embedded the brain-network-based structural constraint of both imaging features into the MSCCA model, which is formulated as Equation (3). We call it the BN-MSCCA.


(3) $$\underset{u,v,w}{max}u^{T}X^{T}Yv+v^{T}Y^{T}Zw+w^{T}Z^{T}Xu$$

$$\begin{array}{l} s.t.\;u^{T}X^{T}Xu\leq 1,\;v^{T}Y^{T}Yv\leq 1,w^{T}Z^{T}Zw\leq 1,\\ ||u|{|}_{1}+||u|{|}_{bn}\leq a_{1},\;||v|{|}_{1}+||v|{|}_{bn}\leq a_{2},\\ ||w|{|}_{1}\leq a_{3} \end{array}$$

Here ||*u*||_*bn*_ and ||*v*||_*bn*_ are the brain-network-based structural penalties, introducing the brain-network-based prior information. Their definitions were given in Equations (4) and (5), respectively.


(4) $$\begin{array}{l} ||u|{|}_{bn}=\;\overset{K}{\underset{k=1}{\sum }}\sqrt{\underset{j\in k}{\sum }u_{j}^{2}}=\overset{K}{\underset{k=1}{\sum }}\;||U^{k}|{|}_{2} \end{array}$$

(5) $$\begin{array}{l} ||v|{|}_{bn}=\;\overset{K}{\underset{k=1}{\sum }}\sqrt{\underset{j\in k}{\sum }v_{j}^{2}}=\overset{K}{\underset{k=1}{\sum }}\;||V^{k}|{|}_{2} \end{array}$$

Specifically, the ROI-based GM volume and d-fALFF features were extracted based on the same AAL template (116 ROIs). We manually grouped these 116 regions into *K* = 15 brain networks (including both left and right hemispheres) according to a previous study (Han et al., 2019). In our work, the cerebellum was divided into two networks (each in one hemisphere), and the whole vermis was treated as a single brain network. Thus, the objective function for BN-MSCCA was rewritten as follows.


(6) $$\begin{array}{l} \underset{u,v,w}{min}-u^{T}X^{T}Yv-v^{T}Y^{T}Zw-w^{T}Z^{T}Xu+\frac{1}{2}{\left\|Xu\right\|}_{2}^{2}\\ +\frac{1}{2}{\left\|Yv\right\|}_{2}^{2}+\frac{1}{2}{\left\|Zw\right\|}_{2}^{2}+\lambda_{1}\alpha {\left\|u\right\|}_{bn}+\lambda_{1}\left(1-\alpha \right){\left\|u\right\|}_{1}\\ +\lambda_{2}\beta {\left\|v\right\|}_{bn}+\lambda_{2}\left(1-\beta \right){\left\|v\right\|}_{1}+\lambda_{3}{\left\|w\right\|}_{1} \end{array}$$

In this function, λ_1_, λ_2_, λ_3_, α, and β are the non-negative tuning parameters. λ_1_, λ_2_, and λ_3_ are used to balance between the penalty and the loss function, whereas α and β are used to balance the brain-network-based and individual ROI-based feature selections for the functional and structural modalities respectively.

### Optimization Algorithm

In this study, we used the alternative iteration algorithm to optimize the BN-MSCCA. To minimize the equation, we take the derivate of the objective function with respect to *u*, *v*, and *w* separately and make them approach zero. Then, we arrive at


(7) $$\begin{array}{l} u={\left(X^{T}X+\;\lambda_{1}\alpha {\bar{D}}_{1}+\;\lambda_{1}\left(1-\alpha \right)D_{1}\right)}^{-1}X^{T}\left(Yv+Zw\right) \end{array}$$

*D*_1_ is a diagonal matrix with the *j*-th diagonal entry being $\frac{1}{\left|u_{j}\right|}$. ${\bar{D}}_{1}$ is a block diagonal matrix of the *k*-th diagonal block as $\frac{1}{2||U^{k}|{|}_{2}}$. Using the same procedure, we can obtain the solution of *v* and *w*:


(8) $$\begin{array}{l} v={\left(Y^{T}Y+\;\lambda_{2}\beta {\bar{D}}_{2}+\;\lambda_{2}\left(1-\beta \right)D_{2}\right)}^{-1}Y^{T}\left(Xu+Zw\right) \end{array}$$

(9) $$\begin{array}{l} w={\left(Z^{T}Z+\;\lambda_{3}D_{3}\right)}^{-1}Z^{T}\left(Xu+Yv\right) \end{array}$$

During each iterative procedure, we first fix *v* and *w* to solve *u*, then fix *u* and *w* to solve *v*, and finally fix *u* and *v* to solve *w*. The process stops until meeting the stopping criterion. Algorithm 1 shows the pseudocode of the BN-MSCCA algorithm.

**Algorithm 1:** BN-MSCCA.

**Require:**
d-fALFF-based features $X={\left[x_{1},\ldots ,x_{n}\right]}^{T}\in {\mathbb{R}}^{n\times p}$, GM volume-based features $Y={\left[y_{1},\ldots ,y_{n}\right]}^{T}\in {\mathbb{R}}^{n\times q}$, *p* = *q* = 116 in our study, diagnosis information $Z={\left[z_{1},\ldots ,z_{n}\right]}^{T}\in {\mathbb{R}}^{n\times r},r=1$ **Ensure**: canonical weights *u*, *v*, *w* Initialization: *u* ∈ ℝ^*p*×1^, *v* ∈ ℝ^*q*×1^, *w* ∈ ℝ^*r*×1^ **While** not converged **do** Calculate the diagonal matrix ${\bar{D}}_{1}$ and *D*_1_; Update $u={\left(X^{T}X+\;\lambda_{1}\alpha {\bar{D}}_{1}+\;\lambda_{1}\left(1-\alpha \right)D_{1}\right)}^{-1}X^{T}\left(Yv+Zw\right)$; Scale *u* so that $||Xu|{|}_{2}^{2}$ = 1; Calculate the diagonal matrix ${\bar{D}}_{2}$ and *D*_2_; Update $v={\left(Y^{T}Y+\;\lambda_{2}\beta {\bar{D}}_{2}+\;\lambda_{2}\left(1-\beta \right)D_{2}\right)}^{-1}Y^{T}\left(Xu+Zw\right)$; Scale *v* so that $||Yv|{|}_{2}^{2}$ = 1; Calculate the diagonal matrix *D*_3_; Update $w={\left(Z^{T}Z+\;\lambda_{3}D_{3}\right)}^{-1}Z^{T}\left(Xu+Yv\right)$; Scale *w* so that $||Zw|{|}_{2}^{2}$ = 1; **End while**

## Experiments and Results

### Experimental Setup

To evaluate the effectiveness of the proposed BN-MSCCA, we chose three closely related methods as the benchmarks. They are SCCA (Witten et al., 2009), MSCCA (Hao et al., 2017), and SCCAR (Du et al., 2019). These three methods could find the associations between GM volume-based and d-fALFF-based features. However, the SCCA ignores the diagnosis information. SCCAR combines the linear regression with SCCA to guide the correlation analysis using the diagnosis information, and the discriminative biomarkers could be detected. Both MSCCA and BN-MSCCA extend the SCCA into three-view condition, so that it could introduce the diagnosis information into model for guiding the association identification; meanwhile, BN-MSCCA further incorporated the brain-network-based structure information as prior.

There are five parameters λ_1_, λ_2_, λ_3_, α, and β in the proposed BN-MSCCA method. The α and β were fixed as 0.5 to balance the brain-network-based and individual ROI-based feature selections for the functional and structural modalities respectively. Such settings simplified the parameter tuning procedure and reduced the time consumption without affecting the performance significantly. The optimal values of λ_1_, λ_2_, and λ_3_ were found by the grid searching strategy during a nested 5-fold cross-validation. For MSCCA and BN-MSCCA, we tuned λ_1_, λ_2_, and λ_3_ in the range of [0.01, 0.1, 1, 10, 100]. As for SCCA and SCCAR, due to the limitation of sparse parameter values by applying the soft-thresholding function (Parkhomenko et al., 2009), we tuned the parameters in the range of [0.01: 0.05: 0.5], according to the strategy in the study of Du et al. (2019). For these four methods, the corresponding optimal parameters were determined by minimizing the differences between training and validating canonical correlation coefficients (CCCs). For each comparison method, the overall procedure was repeated for five times to ensure the robustness of results; meanwhile, the time consumption was acceptable. In addition, the data partition and termination condition were same for all comparison methods. The experiments of all comparison methods were executed on the same software platform.

### Multivariate Association Identification

**Figure 2:**
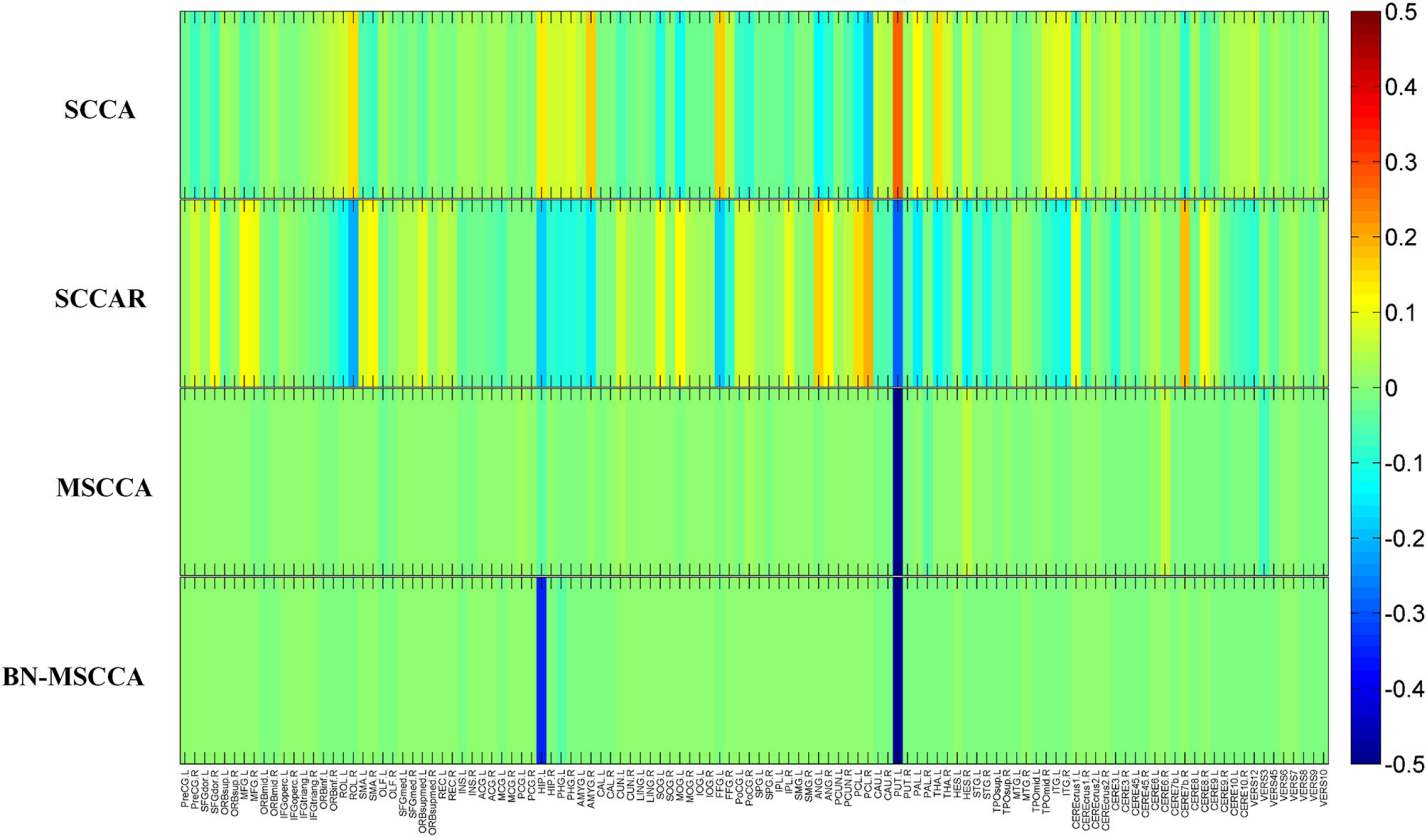
The mean canonical weight *U* of d-fALFF during five times 5-fold cross-validation.

**Figure 3:**
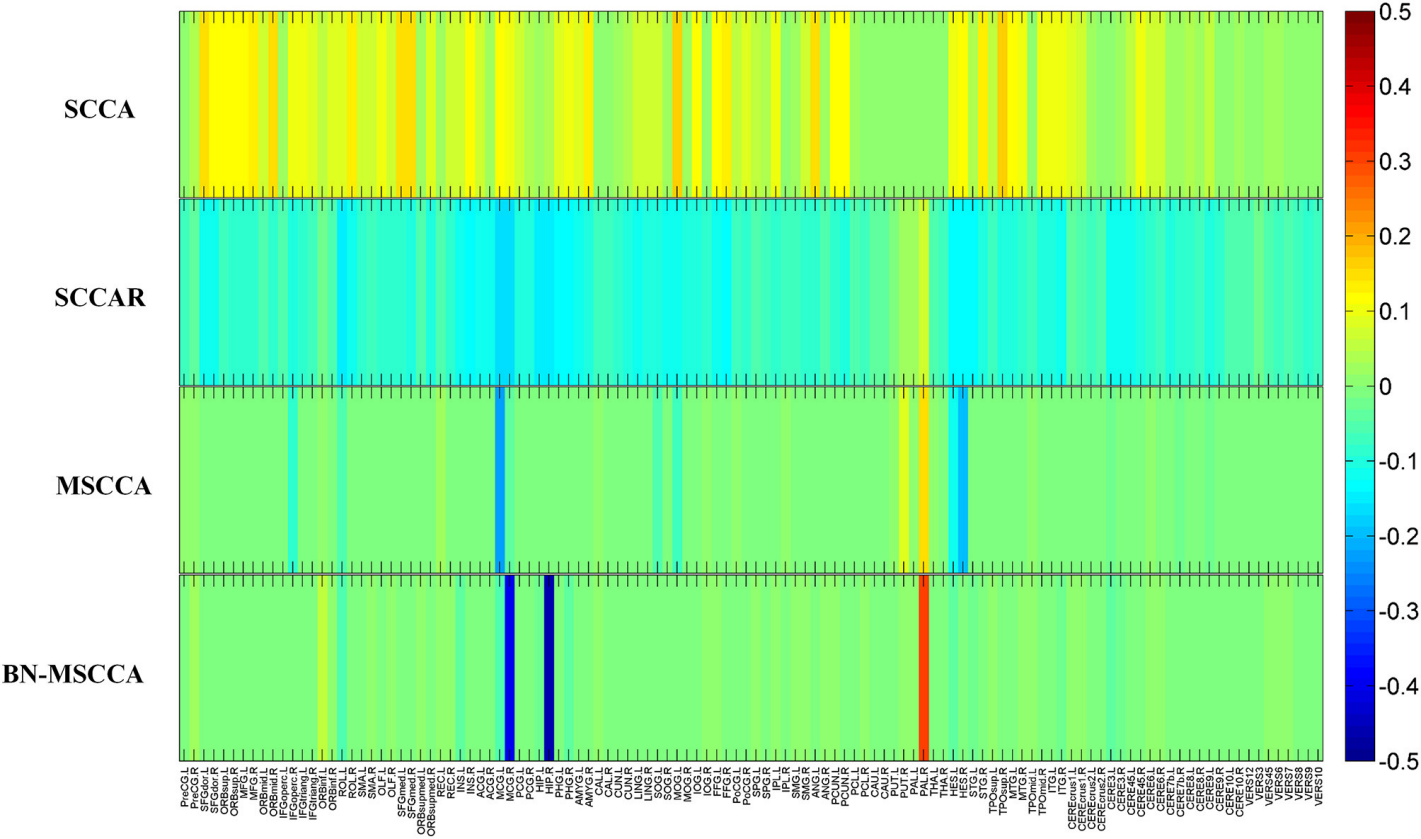
The mean canonical weight *V* of gray matter volume during five times 5-fold cross-validation.

In the field of medical image analysis, the detected imaging biomarkers are of great importance. In this work, we compared the amplitude of the canonical weight, which indicated the importance of the biomarkers. After the cross-validation, the canonical weights were averaged for each ROI. The heatmaps of canonical weights *U* of d-fALFF and *V* of GM volume for different methods are shown in Figures 2, 3, respectively. In these figures, each row stands for the canonical weights for one method, in which the deeper color indicates the features corresponding to the canonical weights more important. From Figures 2, 3, it can be seen that the SCCA and SCCAR methods identified too many signals, which may misguide the subsequent investigation. MSCCA and our proposed method detected more sparse canonical weight patterns than SCCA and SCCAR methods. Moreover, our method obtained more interesting canonical weight patterns compared with the MSCCA. As shown in Figure 2, the largest weight consistently located in left putamen for the MSCCA and BN-MSCCA methods. What is more, the d-fALFF in left hippocampus is also detected by our proposed BN-MSCCA method. Both of them belong to the subcortical network in left hemisphere. As for the canonical weight *V* in Figure 3, the greatest signal in right pallidum is found by the MSCCA and BN-MSCCA methods. Besides this, the right middle cingulate and right hippocampus are also identified by our proposed method. These three ROIs are within the right subcortical network. These results demonstrated that the proposed BN-MSCCA is very promising in finding the biologically meaningful imaging biomarkers by introducing the brain-network-based structural information.

We also compared BN-MSCCA with other methods in terms of CCCs between d-fALFF-based and GM volume-based features. For each method, the CCCs across the cross-validation were averaged for the training and testing data respectively, and their corresponding mean and standard deviation were calculated. From Table 2, we can see that our method achieved the best CCC result in the testing data, which is also more consistent with the CCC result in the training data. This indicates that our method may have better generalization performance compared with the other competing methods.

**Table 2:** Comparison of CCCs on different methods (mean ± standard deviation).

**Method**	**CCC (training)**	**CCC (testing)**
SCCA	0.25 ± 0.02	0.09 ± 0.06
SCCAR	0.26 ± 0.03	0.08 ± 0.06
MSCCA	0.22 ± 0.08	0.11 ± 0.07
BN-MSCCA	0.16 ± 0.03	0.14 ± 0.08

### Classification Setting and Results

To investigate the effectiveness of the identified biomarkers for assisting the diagnosis of SCZ, we performed the classification task based on different feature selection methods. In this study, we compared our BN-MSCCA method with five competing methods, including the method with the original features (without feature selection), the method with two-sample *t*-test feature selection, SCCA, SCCAR, and MSCCA. The details of these methods are summarized as follows.

Original features: In this method, the GM volume-based and d-fALFF-based features were directly concatenated as a feature vector to fit the classifier. Two-sample *t*-test feature selection: Similar to the method with original features, we first obtained the feature vector for each subject. Then, two-sample *t*-test was used to find the most discriminative features. A threshold of *p*-value was set for feature selection. The selected features were used for further model training and testing. The optimal threshold for selecting the features was determined from a set of 10 predefined *p*-values of [0.01–0.1] with the step of 0.01. SCCA: In this method, the GM volume-based and d-fALFF-based features were analyzed using SCCA. According to the absolute value of canonical weights, the top ten features were selected in each imaging modality for the classification. SCCAR: Similarly, the GM volume-based and d-fALFF-based features were analyzed using SCCAR. According to the absolute value of canonical weights, the top ten features were selected for the classification. MSCCA: The GM volume-based and d-fALFF-based features were selected using MSCCA method. Different with SCCA, the diagnosis information was treated as the third type of data to guide the feature selection. We used the features with top ten absolute value of canonical weights in each imaging modality for the classification. BN-MSCCA: The GM volume-based and d-fALFF-based features were selected using BN-MSCCA, with the guidance of diagnosis information for feature selection. And the features corresponding to the top ten absolute value of canonical weights were used to conduct the classifier.

In our study, all the methods used the linear kernel-based support vector machine (SVM) with the same default setting to perform the classification. The 10-fold cross-validation was repeated ten times to ensure the robustness of the model. Finally, the classification performance was evaluated by computing metrics such as the accuracy (ACC), specificity (SPE), sensitivity (SEN), and area under the curve (AUC). As shown in Table 3, we can see that the two-sample *t*-test based method, SCCAR, MSCCA, and BN-MSCCA outperform the method with original features. However, the classification performance of SCCAR and MSCCA methods are slightly worse than that of two-sample *t*-test based method, as the latter one is directly designed for finding the discriminative features between two classes. Apparently, our BN-MSCCA method achieves the best performance, and also has the increments of 5.33%, 5.01%, 5.63%, and 6.77% on ACC, SEN, SPE, and AUC respectively, compared with the two-sample *t*-test based method. Overall, we can conclude from these results that the GM volume-based and d-fALFF-based features are effective for the classification of SCZ. After using the feature selection, the performance could be improved. In addition, our proposed BN-MSCCA particularly takes both the diagnosis information and the brain-network-based structural information into consideration, achieving the best classification performance.

**Table 3:** Comparison of classification performance on different feature selection methods (mean ± standard deviation).

**Method**	**ACC (%)**	**SEN (%)**	**SPE (%)**	**AUC (%)**
Original features	62.98 ± 7.96	63.28 ± 12.04	62.62 ± 10.80	67.90 ± 8.30
Two-sample *t*-test	65.17 ± 7.24	64.72 ± 10.70	65.63 ± 10.34	69.77 ± 7.36
SCCA	62.04 ± 8.02	62.39 ± 11.30	61.73 ± 12.13	66.20 ± 8.27
SCCAR	64.92 ± 7.33	63.89 ± 11.05	65.95 ± 9.69	68.63 ± 8.03
MSCCA	64.47 ± 7.42	64.32 ± 11.82	64.57 ± 10.69	70.54 ± 8.20
BN-MSCCA	**70.50** **±7.43**	**69.73** **±10.31**	**71.26** **±10.76**	**76.54** **±7.46**

*Bold values indicate the best results*.

### Correlation With PANSS Score

**Figure 4:**
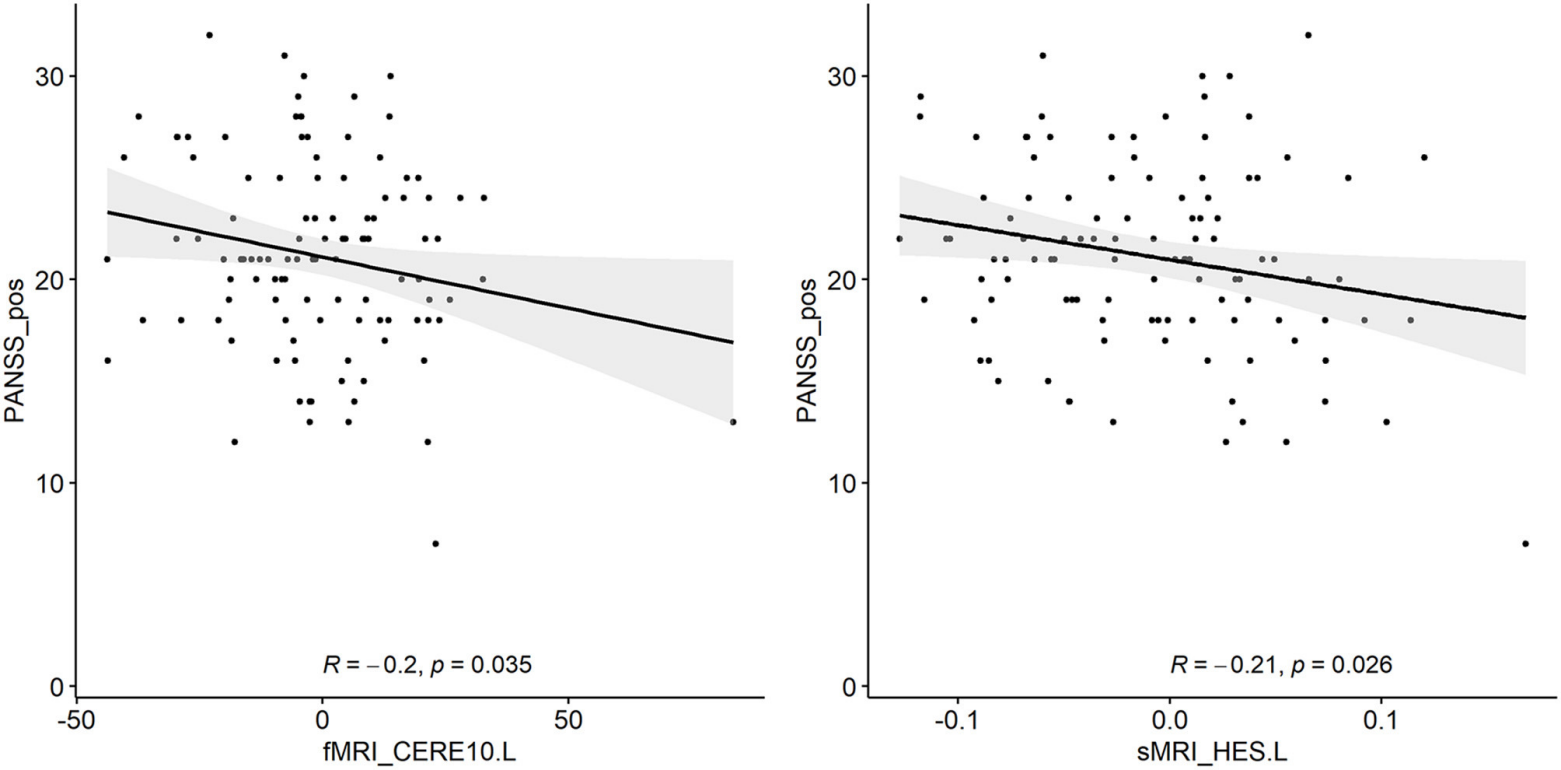
Two detected biomarkers (adjusted values) which have significant correlations with the positive symptom score of PANSS.

The correlation analysis between the detected biomarkers and the PANSS score has been regarded as a proof for the effectiveness of the feature selection in this SCZ research. We used the adjusted features to conduct the correlation with the PANSS scores based on 108 patients with SCZ, whose PANSS scores were available and complete for this analysis. Figure 4 shows that significant correlations exist between the PANSS scores and two of the detected biomarkers respectively. It is obvious that the d-fALFF of left cerebellum shows a significant negative correlation with the positive symptom score of PANSS (*R* = −0.2, *p* = 0.035). Additionally, the gray matter volume of left heschl is negatively correlated with positive symptom score of PANSS at a significant level (*R* = −0.21, *p* = 0.026).

## Discussion

In this section, we summarized the main idea and contributions of this study and further discussed the top 10 identified brain regions of d-fALFF and gray matter volume respectively, following with the investigation about their pairwise correlations. Then, both the classification with the identified biomarkers and the correlation between PANSS score and the detected biomarkers are analyzed. Finally, the limitations of our method and the potential future study directions are presented.

### Main Idea and Contributions

In this study, a brain-network-constrained multi-view SCCA was proposed. It has been demonstrated that the proposed method has significantly improved performance for the identification of brain structural and functional biomarkers, compared with the other competing methods. The main idea and key contributions of this study are summarized as follows: (1) a novel model was proposed to jointly analyze the d-fALFF, gray matter volume, and diagnosis information for the identification of SCZ-related biomarkers; (2) the brain-network-based structural constraint was introduced into the model, so that the detected biomarkers were interpretable; (3) the experiments were performed on 191 patients with SCZ and 191 matched healthy controls, and the proposed method achieved superior performance for the biomarker detection, compared with the other methods; (4) the effectiveness of detected biomarkers was further verified on two subsequent analysis tasks, including the SCZ-HC classification and the PANSS correlation analysis. The results proved the potential usage of these biomarkers for the clinical applications. Overall, the proposed method would be a powerful alternative method for multimodal analysis. In addition, the findings in this study could be supplementaries and verifications to the exploration of biomarkers for SCZ.

### Top-10 Selected Brain Regions of d-fALFF

**Figure 5:**
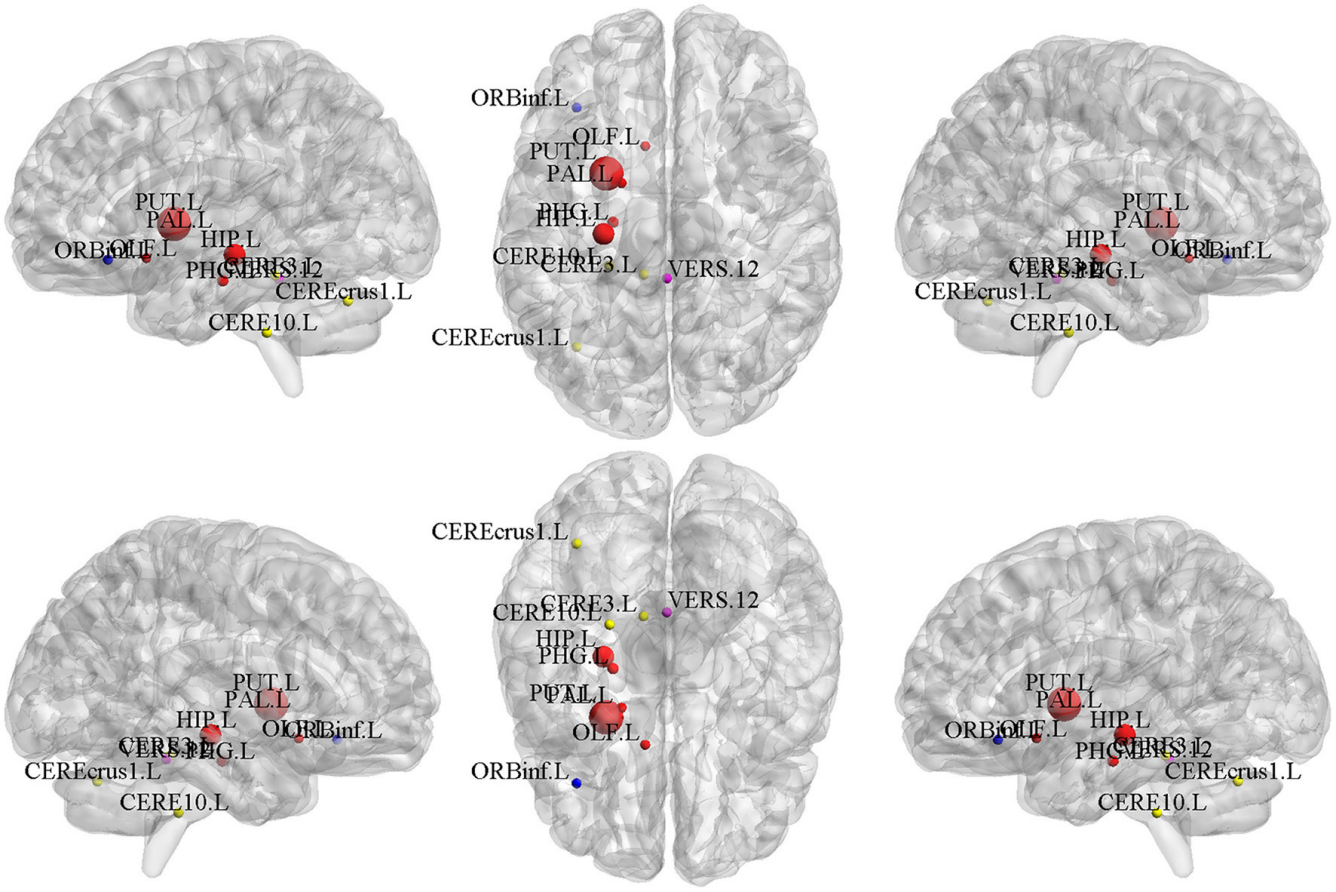
The top 10 ROIs of d-fALFF selected by BN-MSCCA (different colors denote different brain networks).

We calculated the mean values of the canonical weights across the five times 5-fold cross-validation to select the top brain regions of d-fALFF. The top ten ROIs are shown in Table 4. We also visualized these top-10 selected regions in Figure 5. According to Table 4 and Figure 5, we observed that multiple detected regions belong to a certain brain network. For example, five detected regions are within the left subcortical network, including the putamen, hippocampus, parahippocampal, pallidum, and olfactory gyrus in left hemisphere. Previous studies about SCZ have demonstrated the increased functional connectivities between certain subcortical regions and cortical ROIs, showing the important role of the subcortical network in SCZ (Zhang et al., 2012). And the cerebellum might be another key brain region involved in the cognitive function. A study has suggested the functional abnormalities of the cerebellum in a cerebellar-subcortical-cortical loop in the brains of SCZ patients, and it may be the underlying mechanism of SCZ (Zhuo et al., 2018). A total of three ROIs which located in left cerebellum were found in our study, which might be support for this previous finding. The above findings have verified the effectiveness of BN-MSCCA for identifying the interpretable biomarkers of SCZ. With the help of the L1-norm, the proposed method also detected some individual-level SCZ-related ROIs, such as vermis and the left inferior frontal gyrus, which is also consistent with the findings in the previous studies (Jeong et al., 2009,Collin, 2011).

**Table 4:** Top 10 ROIs of d-fALFF identified by our method.

**ROI**	**Related brain network**	**Weight**
PUT.L	SN.L	0.72464
HIP.L	SN.L	0.354
PHG.L	SN.L	0.04529
CEREcrus1.L	CN.L	0.014084
PAL.L	SN.L	0.012935
CERE10.L	CN.L	0.012429
CERE3.L	CN.L	0.0094317
VERS12	VN	0.0060698
ORBinf.L	ATN.L	0.0053477
OLF.L	SN.L	0.0050575

*R, right; L, left; PUT, putamen; HIP, hippocampus; PHG, parahippocampal gyrus; CEREcrus, cerebellum_crus; PAL, pallidum; CERE10, cerebellum_10; CERE3, cerebellum_3; VERS12, vermis_1_2; ORBinf, inferior orbitofrontal cortex; OLF, olfactory; SN, subcortical network; CN, cerebellum network; VN, vermis network; ATN, attention network*.

### Top-10 Selected Brain Regions of Gray Matter Volume

**Figure 6:**
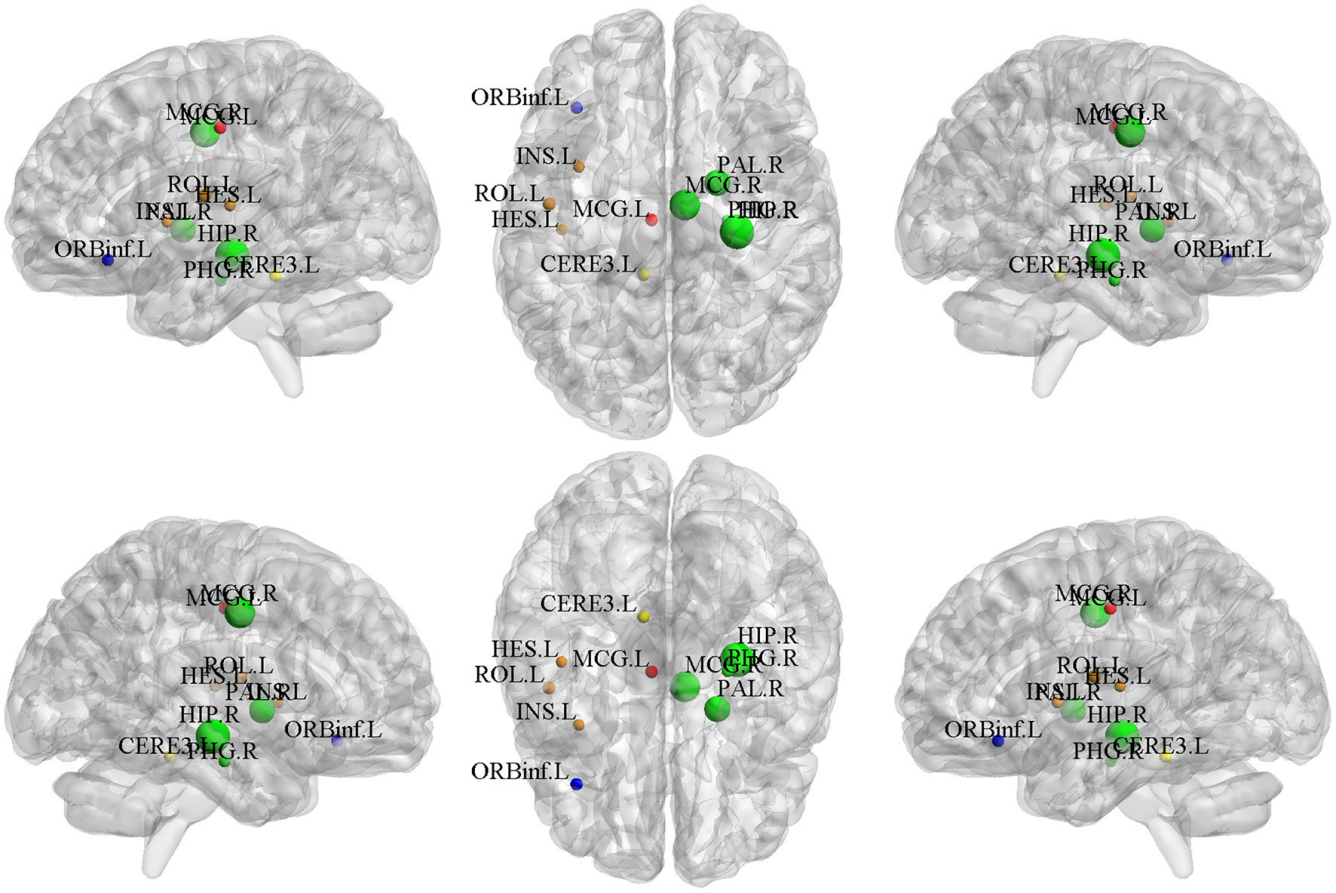
The top 10 ROIs of gray matter volume selected by BN-MSCCA (different colors denote different brain networks).

The top-10 selected brain regions of gray matter volume based on their respective average canonical weights are shown in Table 5. Accordingly, four ROIs in right subcortical network and three ROIs in left auditory network are detected as the most important biomarkers, proving the effectiveness of introducing the brain-network-based structural constraint. The left middle cingulate gyrus, left inferior frontal gyrus, and left cerebellum were also detected in the GM volume-based features, which is prompted by the L1-norm. Figure 6 shows the visualization of these top 10 selected regions. As can be seen in Table 5 and Figure 6, we obtained consistent results with the previous studies about these most important ROIs for SCZ (Witthaus et al., 2009; Kubera et al., 2014; Krause and Pogarell, 2017; He et al., 2019).

**Table 5:** Top 10 ROIs of gray matter volume identified by our method.

**ROI**	**Related brain network**	**Weight**
HIP.R	SN.R	0.46577
MCG.R	SN.R	0.39399
PAL.R	SN.R	0.30736
ROL.L	AUN.L	0.060209
MCG.L	SN.L	0.052595
ORBinf.L	ATN.L	0.047987
CERE3.L	CN.L	0.045651
INS.L	AUN.L	0.038043
PHG.R	SN.R	0.035748
HES.L	AUN.L	0.032479

*R, right; L, left; HIP, hippocampus; MCG, middle cingulate gyrus; PAL, pallidum; ROL, rolandic operculum; ORBinf, inferior orbitofrontal cortex; CERE3, cerebellum_3; INS, insular; PHG, parahippocampal gyrus; HES, heschl; SN, subcortical network; AUN, auditory network; ATN, attention network; CN, cerebellum network*.

### Refined Correlation Analysis

**Figure 7:**
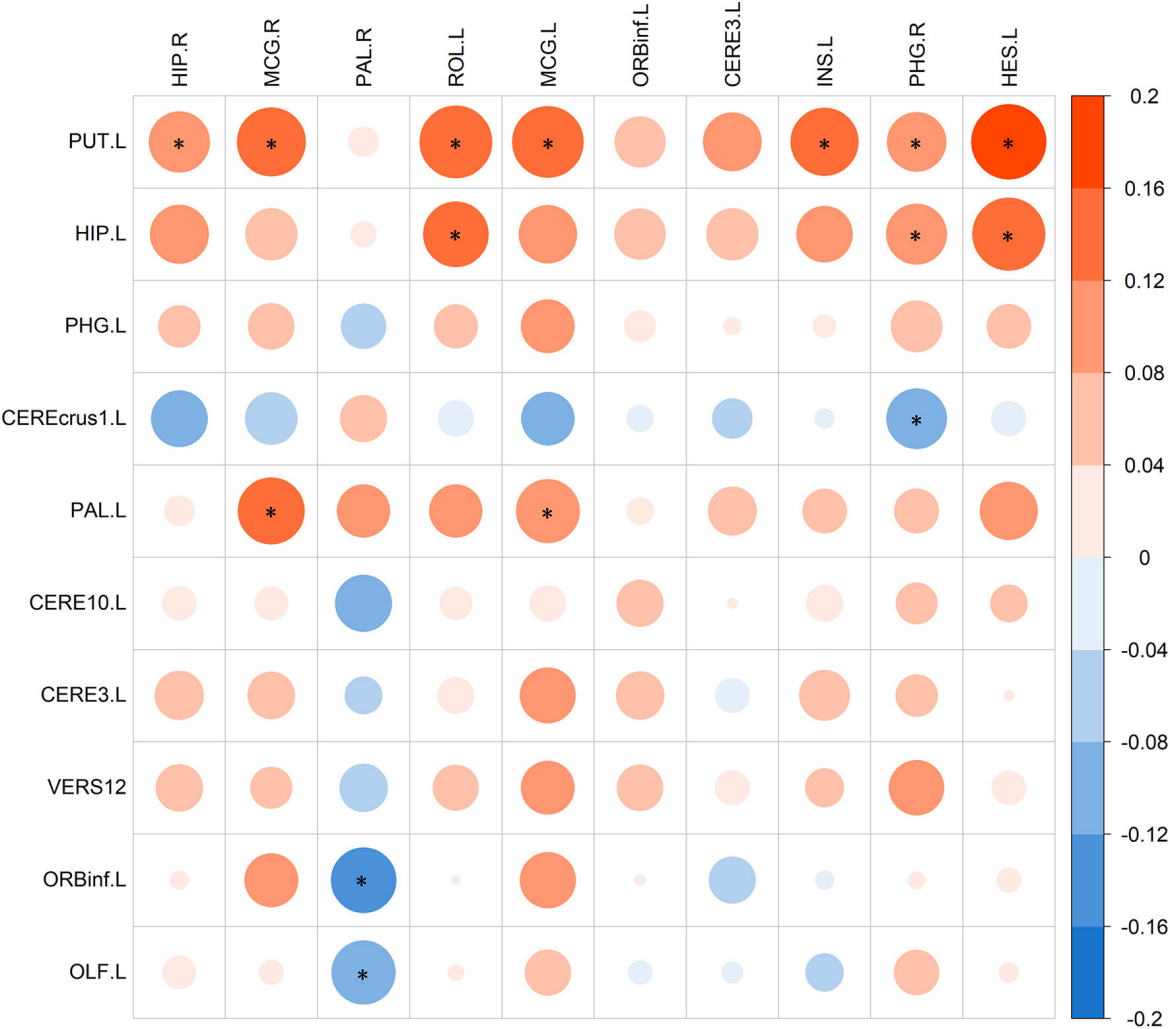
The pairwise correlations between top 10 ROIs of d-fALFF (column) and top 10 ROIs of gray matter volume (row). Here * denotes *p* < 0.05.

After identifying SCZ-related biomarkers for each single modality, we further conducted a refined correlation analysis between d-fALFF-based and GM volume-based biomarkers to explain their relationships. We here present the pairwise correlation results between top 10 ROIs of d-fALFF and top 10 ROIs of gray matter volume. Figure 7 shows the heatmap of this correlation analysis of each pair, where circles labeled with “^*^” indicate that the correlations between the d-fALFF and gray matter volume of their corresponding regions are significant (*p* < 0.05). As shown in Figure 7, when looking horizontally, the d-fALFF of left putamen is significantly correlated with most (seven out of ten) of the GM volume-based biomarkers. The d-fALFF in left hippocampus is positively correlated with three brain regions of gray matter volume (left rolandic operculum, right parahippocampal, and left heschl gyrus) at significant level. When looking vertically, six regions of gray matter volume (bilateral middle cingulate, right pallidum, left rolandic operculum, right parahippocampal, and left heschl gyrus) are significantly correlated with at least two regions of d-fALFF. These pairwise correlation results show that our proposed method could identify the brain regions where the brain function and structure are significantly associated with each other, and these significant correlations might reflect the abnormal brain regions of SCZ.

### SCZ-HC Classification and PANSS Correlation

By now, we have selected the SCZ-related ROIs of d-fALFF and gray matter volume respectively based on our method. To investigate the effectiveness of their potential clinical applications, we performed two subsequent analyses, including the SCZ-HC classification and the correlation with PANSS score. According to the classification results, these two types of features could classify the SCZ from the HC with a reasonable accuracy. By performing the feature selection, we also found that the most discriminative features were retained and the redundant features were discarded, which helped achieve significant improvements in the classification performance. Multiple studies have proved SCZ is a disorder with brain network abnormalities (Rubinov and Bullmore, 2013; Li et al., 2019). The detection of such brain network abnormalities could help capture the different patterns between SCZ and HC. Thus, various studies performed the SCZ-HC classification using the brain network-based measurements, which depicted the abnormal alterations of brain functional or structural network (Han et al., 2019; Lei et al., 2020b). Our method can take both the diagnosis information and the brain-network-based prior information into consideration for the selection of the most discriminative features. The biomarkers detected by the proposed method have strong discriminative power, and the classification performance outperforms all comparison methods.

We also conducted the PANSS correlation analysis based on the detected biomarkers. Two significant negative correlations were found in our study, which included the correlation between the d-fALFF of cerebellum and the positive symptom score of PANSS, and the correlation between the gray matter volume of left heschl and the positive symptom score of PANSS. Interestingly, previous studies have proved the same negative correlation trend between the positive symptom score of PANSS score and these two brain regions in SCZ (Narayanaswamy et al., 2015; Du et al., 2017), demonstrating the reasonability of our findings. Here only two detected biomarkers showed significant correlation with the positive symptom score of PANSS. The potential reason may be that the diagnosis information was used as the target to guide the canonical correlation in this study, which might lead to the detected biomarkers not specific to the PANSS score.

### Limitations and Future Directions

Based on the above experimental results and discussion, we could conclude that our proposed BN-MSCCA has a great capability for the biomarker identification. However, there are also some limitations in this study. First, only two imaging modalities were included in this work. In fact, SCZ is a complex and multi-factor induced disease. The other types of data, such as gene and gut microbiome, were also investigated for SCZ (Guan et al., 2021; Li et al., 2021). These different modalities could provide useful and complementary information, which would be considered in our future work. Second, due to the proposed method is based on the MSCCA, it requires that the identified d-fALFF-based biomarkers should be correlated with GM volume-based biomarkers and diagnosis information simultaneously. Thus the modality-specific correlation and its corresponding biomarkers would be overlooked, which might be also valuable for the understanding of the disease. Third, recent studies have proved that SCZ is a heterogeneous disease comprising various symptoms, which could be divided into multiple subtypes (Chand et al., 2020). However, only two diagnostic classes were considered in this work, ignoring the different patterns of abnormalities among different subtypes of patients. Our future direction includes exploring the biomarkers which are oriented to a specific subtype of SCZ, aiming for the accurate diagnosis and treatment of this disease. Fourth, we only used a specific AAL atlas, which may limit the capability of biomarker detection. Future studies should also consider other widely used atlases for feature extraction, such as Power 264 atlas (Power et al., 2011), exploring the influences of different atlases on BN-MSCCA.

## Conclusion

In this study, we developed a brain-network-constrained multi-view SCCA method namely BN-MSCCA, which could uncover the brain structural and functional associations and identify the potential biomarkers for SCZ. The proposed BN-MSCCA could leverage the inter-modality associations to better find the disease-related multimodal neuroimaging biomarkers, which is achieved by performing the multi-view sparse canonical correlation analysis among brain structural features, functional features, and diagnosis information simultaneously. Moreover, the identified biomarkers were encouraged to locate in multiple predefined brain networks. Thus more biologically interpretable results could be achieved, which was guaranteed by incorporating the brain-network-based structural constraint.

The proposed method was validated on a SCZ dataset, with the aim of mining the relationship between d-fALFF-based features and GM volume-based features and further finding the SCZ-related biomarkers. Compared with the SCCA, SCCAR, and MSCCA method, the BN-MSCCA could not only identify more sparse and meaningful canonical weight patterns, but also obtain the larger testing CCC. Furthermore, the detected biomarkers were evaluated by the subsequent classification and correlation analysis tasks for validating the effectiveness of their clinical applications. Experimental results showed that our method could identify more discriminative biomarkers, achieving the superior classification performance to other competing strategies for feature selection. Moreover, the significant negative correlations were found between the positive symptom score of PANSS and two of the identified biomarkers respectively, demonstrating the promising application of these biomarkers in discovering the severity of SCZ symptoms.

## Data Availability Statement

The original contributions presented in the study are included in the article/supplementary material, further inquiries can be directed to the corresponding author/s.

## Ethics Statement

The studies involving human participants were reviewed and approved by the Human Ethics Committee of the First Affiliated Hospital of Zhengzhou University, China (Approval No. 2016-LW-17). Written informed consent to participate in this study was provided by the participants' legal guardian/next of kin. Written informed consent was obtained from the individual(s), and minor(s)' legal guardian/next of kin, for the publication of any potentially identifiable images or data included in this article.

## Author Contributions

PS and YW designed the study and revised the manuscript. YW provided guidance and suggestions for the study. PS performed experiments and drafted the manuscript. XS, XY, and SW collected the imaging and clinical data, and provided these data along with the description of the data. All authors read and approved the submitted manuscript.

## Funding

This study was supported by the National Natural Science Foundation of China (U1504606, U21A20367, and 81971253), China Postdoctoral Science Foundation (2016T90679 and 2015M582201), Key Research Projects of Henan Higher Education Institutions (20A510009), Science and Technology Development Plan of Henan Province (172102310270), and Zhong Yuan Technological Innovation leading Talents (204200510019).

## Conflict of Interest

The authors declare that the research was conducted in the absence of any commercial or financial relationships that could be construed as a potential conflict of interest.

## Publisher's Note

All claims expressed in this article are solely those of the authors and do not necessarily represent those of their affiliated organizations, or those of the publisher, the editors and the reviewers. Any product that may be evaluated in this article, or claim that may be made by its manufacturer, is not guaranteed or endorsed by the publisher.
